# Small-RNA sequencing identifies dynamic microRNA deregulation during skeletal muscle lineage progression

**DOI:** 10.1038/s41598-018-21991-w

**Published:** 2018-03-09

**Authors:** David Castel, Meryem B. Baghdadi, Sébastien Mella, Barbara Gayraud-Morel, Virginie Marty, Jérôme Cavaillé, Christophe Antoniewski, Shahragim Tajbakhsh

**Affiliations:** 1 Stem Cells and Development, Department of Developmental & Stem Cell Biology, Institut Pasteur, Paris, 75015 France; 2 CNRS UMR 3738, Institut Pasteur, Paris, 75015 France; 3 Sorbonne Universités, UPMC, University of Paris 06, IFD-ED 515, 4 Place Jussieu, Paris, 75252 France; 4 Laboratoire de Biologie Moléculaire Eucaryote, Centre de Biologie Intégrative (CBI), Université de Toulouse, CNRS, UPS, 31000 Toulouse, France; 5 Sorbonne Universités, Université Pierre et Marie Curie (UPMC), CNRS, Institut de Biologie Paris Seine (IBPS), Developmental Biology Department, Paris, France; 6 Sorbonne Universités, Université Pierre et Marie Curie (UPMC), CNRS, Institut de Biologie Paris Seine (IBPS), ARTbio Bioinformatics Analysis Facility, Paris, France; 7 Present Address: Département de Cancérologie de l’Enfant et de l’Adolescent & UMR8203 “Vectorologie et Thérapeutiques Anticancéreuses”, CNRS, Gustave Roussy, Univ. Paris-Sud, Université Paris-Saclay, 94805 Villejuif, France

## Abstract

Skeletal muscle satellite cells are quiescent adult resident stem cells that activate, proliferate and differentiate to generate myofibres following injury. They harbour a robust proliferation potential and self-renewing capacity enabling lifelong muscle regeneration. Although several classes of microRNAs were shown to regulate adult myogenesis, systematic examination of stage-specific microRNAs during lineage progression from the quiescent state is lacking. Here we provide a genome-wide assessment of the expression of small RNAs during the quiescence/activation transition and differentiation by RNA-sequencing. We show that the majority of small RNAs present in quiescent, activated and differentiated muscle cells belong to the microRNA class. Furthermore, by comparing expression in distinct cell states, we report a massive and dynamic regulation of microRNAs, both in numbers and amplitude, highlighting their pivotal role in regulation of quiescence, activation and differentiation. We also identify a number of microRNAs with reliable and specific expression in quiescence including several maternally-expressed miRNAs generated at the imprinted *Dlk1-Dio3* locus. Unexpectedly, the majority of class-switching miRNAs are associated with the quiescence/activation transition suggesting a poised program that is actively repressed. These data constitute a key resource for functional analyses of miRNAs in skeletal myogenesis, and more broadly, in the regulation of stem cell self-renewal and tissue homeostasis.

## Introduction

Adult skeletal muscles can regenerate robustly to confront mild and severe lesions induced by exercise or trauma. This extraordinary regenerative capacity occurs largely through the mobilization of resident muscle satellite (stem) cells. These cells are quiescent in resting muscle and can activate, proliferate and differentiate to form new muscle fibres^1^. During lineage progression, a subset of proliferating satellite cells self-renew in their niche by reversibly exiting the cell cycle. Therefore, skeletal myogenesis is a tractable model to study the regulation of quiescence, self-renewal and differentiation.

Micro-RNAs (miRNAs) are ~22-nucleotide long non-coding RNAs that participate in post-transcriptional regulation of gene expression through mRNAs decay or translational repression^2^. Stem-loop structured pre-miRNAs are excised from primary miRNAs and exported to the cytoplasm. Further excision of the loop of pre-miRNA by *Dicer* gives rise to miRNA/miRNA* duplexes. Single-strand miRNAs are then loaded within the RNA-Induced Silencing Complex and guide RISC to complementary sequences in 3′UTR of target mRNAs^3,4^. The miRNA pathway has been shown to play a major role in cell specification and differentiation in many organisms, and also more broadly in organism development, tissue homeostasis. Germ line loss of *Dicer* is lethal at gastrulation, demonstrating an absolute requirement of miRNAs for mouse development^5^. Other studies have demonstrated the specific requirement of miRNAs in ES cells and tissue specific stem cells^6,7^.

A set of miRNAs is associated with differentiation of skeletal muscle cell lines^8–10^. These so-called myomiRs, are induced by the myogenic transcription factors Myod and Myogenin (Myog), and can promote muscle differentiation *in vitro*. Conditional deletion of *Dicer* in myogenic progenitors expressing *Myod* in embryos (*Myod*^*Cre*^; *Dicer*^*flox*^) results in muscle hypoplasia and perinatal lethality^11^ supporting an essential role of miRNAs in muscle development. This role was further dissected during muscle formation and homeostasis and repair in experiments using a *Dicer* conditional KO allele in conjunction with the satellite cell Cre recombinase driver mouse *Pax7-Cre*^*ERT2*^. In this case, satellite cells exiting from quiescence underwent apoptosis, thus resulting in failed regeneration after muscle injury^12^. The initial finding that some miRNAs were expressed in a tissue-specific fashion was confirmed in a study showing that miR-1, miR-122a and miR-124a expression is restricted to striated muscle, liver and brain, respectively^13^, whereas 30 miRNAs are enriched or specifically expressed in skeletal muscle^14^. Interestingly, myomiRs appear to have either uniform expression throughout the muscle (miR-1 and miR-133a)^15,16^, or are enriched in slow-twitch, type I muscles (miR-206, miR-208b and miR-499)^17,18^. In addition, several candidate miRNAs that regulate the quiescence-activation transition in satellite cells were identified, most notably miR-27b^19^, miR-489^12^, miR-31^20^ and miR-195/497^21^.

As previous quantitative and differential data obtained using RT-qPCR or miRNA-microarrays were limited to the quantification of known molecules, we performed an unbiased analysis of small-RNA profiles from stem to differentiated cells in adult myogenesis. Our data provide a key resource for functional studies of the involvement of small-RNAs - including miRNAs, in skeletal muscle, and more broadly in the regulation of stem cell self-renewal and tissue homeostasis.

## Results

### Small RNA profiling during lineage progression of muscle satellite cells

**Figure 1:**
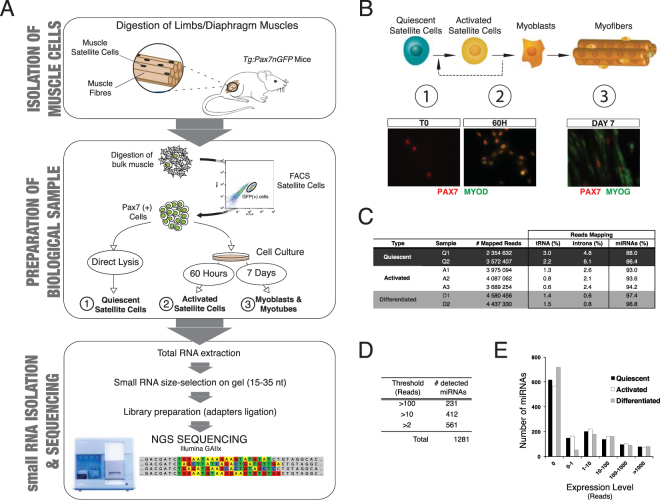
Unbiased identification of stage specific small RNAs during lineage progression from muscle stem cells. (**A**) Quiescent satellite cells were isolated after digestion of resting limb muscles and diaphragm from adult *Tg:Pax7-nGFP* mice by FACS using GFP fluorescence. An aliquot was cultured *in vitro* for 60 h or 7 days, and the remainder was lysed directly for RNA extraction. After size selecting 15–35 nucleotides small RNAs on a polyacrylamide gel, sequencing libraries were prepared and analysed. (**B**) Schematic representation of lineage progression in adult skeletal muscle. Quiescent, activated and differentiated samples are represented. Immuno-fluorescence images confirmed the cellular identity of the 3 populations (i) quiescent satellite cells: Pax7(+), Myod(−); Activated satellite cells/myoblasts: Pax7(+), MyoD(+); Differentiated muscle cells: Pax7(−) Myog(+). Note the presence of rare self-renewing “reserve cells” expressing Pax7 in the differentiated sample. (**C**) Sequenced small RNA corresponded overwhelmingly to miRNAs in all 3 samples, and showed low contamination by degraded tRNA. Despite the inclusion of the 25–32 nt size range in the analysis, no piRNA sequences were detected, whereas reads mapping to intronic regions were identified in particular in the quiescent samples (>5% reads). (**D**) 412 and 231 miRNAs were detected in at least one sample type more than 10 or 100 times, respectively. (**E**) Frequency histogram displaying the miRNAs distribution according to their expression levels in all 3 samples highlight their large dynamic range in expression.

To identify small RNAs expressed during muscle lineage progression, we sequenced small-RNAs from total RNA of quiescent (freshly isolated), activated (60 h in culture) and differentiated (7 days in culture) myogenic cells. Quiescent satellite cells were isolated by fluorescence-activated cell sorting (FACS) from adult transgenic *Tg:Pax7-nGFP*^22^ mouse limb muscles and subsequently lysed for RNA extraction or *in vitro* culture (Fig. 1A). Immunological staining confirmed that freshly isolated cells expressed Pax7 whereas Myod expression was undetectable (Fig. 1B). Sixty hours after plating in proliferation medium, myoblasts expressed Myod and retained Pax7 expression, whereas the latter was largely lost after 7 days in culture when the majority of the cells differentiated.

After RNA extraction, small RNAs were size-selected on gel (15–35 nucleotides), cloned and sequenced on a Illumina GAIIx platform. For each time point, 2 to 3 biological replicates yielded on average 3.8 million reads [2.3–4.4] that were mapped to Mm9 genome (Fig. 1C). Further alignment of reads to tRNA and mRNA sequences revealed a low level of contamination from degraded tRNA sequences (0.6 to 3%), whereas mRNA sequences were barely detectable, thereby confirming the quality of the samples. As expected, alignment against mature miRNA sequences (miRBase Release 19) highlighted the fact that the vast majority of sequences corresponded to miRNAs (93% [86–97%]) and marginally to intronic sequences (3% [0.6–6%]). Interestingly, myomiRs represented only 3% of miRNAs expressed in quiescent satellite cells, but as high as 74% of miRNAs expressed in differentiated cells (Supplementary Fig. S1A). Other classes of small RNAs and in particular piRNAs were not detected in our samples. We subsequently focused on the expression profiles of miRNAs.

### miRNAs are widely expressed throughout the muscle lineage

Following more detailed examination of the miRNA expression data, we observed that out of the 1,281 miRNA sequences used as reference for alignment at the time of the analysis (miRbase r19), 412 (32%) mature miRNAs with an average of more than 10 reads were detected in one biological condition, demonstrating a wide miRNA repertoire expressed in the adult muscle lineage (Fig. 1D). Furthermore, a very large expression range was observed among these miRNAs, with more than 100 miRNAs showing more than 1000 reads in one condition (Fig. 1E). The distribution of the number of expressed miRNAs according to their expression level was closely comparable for each of the quiescent, activated and differentiated biological states, suggesting an overall similar miRNA abundance during myogenic commitment. However, examination of the relative abundance of the few miRNAs highly expressed during quiescence in the other two conditions pointed to dramatic changes in expression of distinct miRNAs (Supplementary Fig. S1B). This observation underscored the importance of robust normalization of the datasets to avoid skewing of the expression profiles as a result of the high expression of a limited number of miRNAs.

### miRNAs expression profiles show dynamic regulation during lineage progression

Following normalization, hierarchical clustering regrouped the samples according to each biological condition (Pearson correlation coefficient R^2^ > 0.92 among replicates) demonstrating the robustness of the datasets (Supplementary Fig. S1C, and Supplementary Table S1). We confirmed the increase in expression of myomiRs (*i.e*. miR-1, miR-133, miR-206 and miR-378) during myogenic commitment (Supplementary Fig. S2A–E), as well as the expression of quiescence associated miR-195 and miR-489 previously reported (Supplementary Fig. S2F–G)^8,10,12,21^. However, we did not recapitulate the expression profiles of miR-27b and miR-31 that were reported to be upregulated in Pax3-positive quiescent satellite cells isolated from abdominal and diaphragm muscles (Supplementary Fig. S2H–I)^19,20^. Our data are in agreement with expression profiles previously published for these miRNAs using RT-qPCR of quiescent and activated satellite cells from limb muscles^12^.

**Figure 2:**
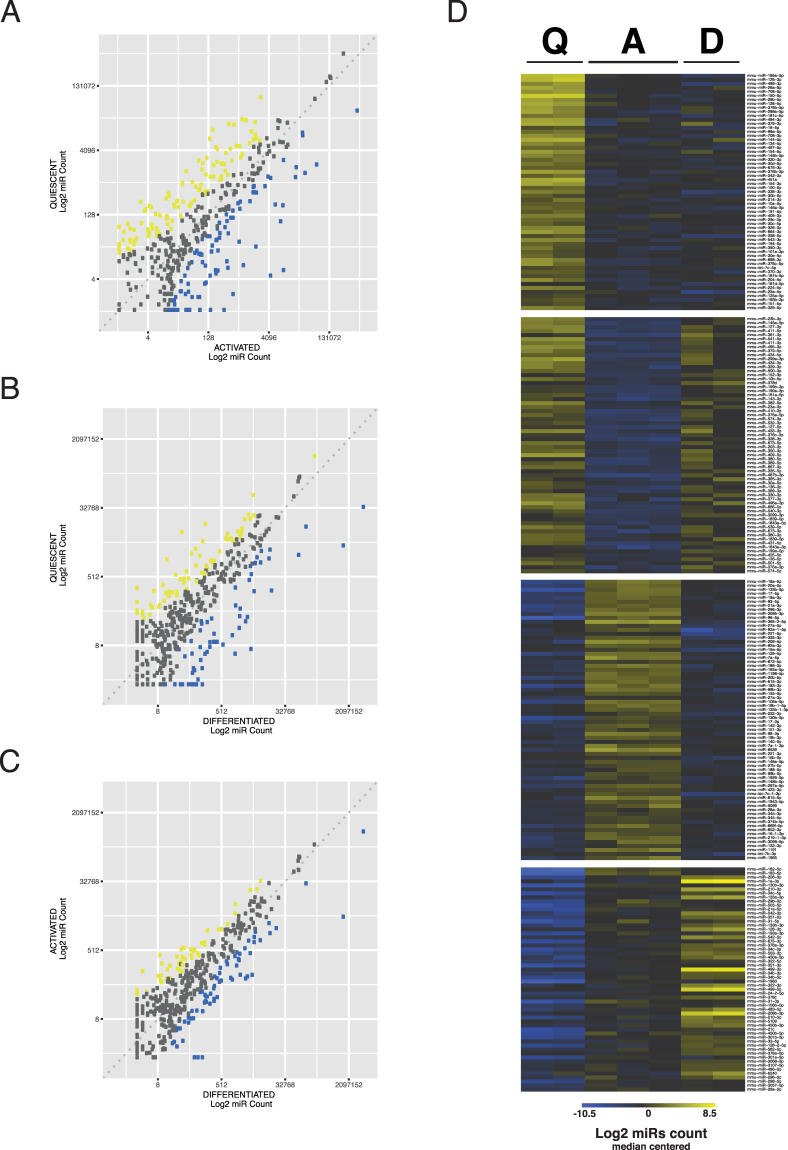
Identification of differentially expressed miRNAs during myogenic lineage progression. (**A**) Scatter plot of miRNA expression level in Quiescent *vs*. Activated, (**B**) Quiescent *vs*. Differentiated and (**C**) Activated *vs*. Differentiated myogenic cells. Results are presented as the median of log transformed normalized counts for each miRNA. Out of 412 miRNAs detected, 249 showed a modulation that reached statistical significance in the 3 pairwise comparisons (corrected *p-*value ≤ 0.001). Statistically significant up- or down-regulated miRNAs were coloured in yellow and blue, respectively. (**D**) Heatmap presenting 4 classes of differentially expressed miRNAs identified by K-means clustering. MicroRNAs are involved in the regulation of all processes – quiescence, activation and self-renewal and differentiation, and a large number of miRNAs with expression specific of one particular state were identified. High expression is coloured in yellow, whereas low expression is blue as in previous panels.

We then conducted a differential analysis between quiescent satellite cells, activated and differentiated myogenic cells. Out of the 412 miRNAs that were expressed, we identified 249 differentially expressed miRNAs in the 3-pairwise comparisons (corrected *p*-value < 0.001): 209 between quiescent and activated, 126 between quiescent and differentiated, and 110 between activated and differentiated muscle cells (Fig. 2A–C). Thus, dynamically expressed micro-RNAs appear to be involved in the regulation of each of the tested cell states. Importantly, we observed that the majority of differential miRNA expression patterns were related to the transition from quiescence to activation (Supplementary Fig. S3).

We then grouped the differentially expressed miRNAs according to their expression profiles using K-means clustering which reveals 4 classes (Fig. 2D and Supplementary Table S2). The first consisted of 59 miRNAs whose expression was found to be associated specifically with quiescence. The second and third clusters comprised miRNAs either expressed during activation, or conversely silenced in this cell state; they represented 70 and 64 miRNAs, respectively. Finally, the last cluster was composed of miRNAs showing an increase in expression during commitment and differentiation, among which were the myomiR class. Overall, the most important transition was between quiescence and activation, where more than half of the differentially expressed miRNAs identified were specific to these states. This finding highlights the concerted role that miRNAs play during the regulation in this transition.

### Dynamic regulation of miRNAs during regenerative myogenesis *in vivo*

**Figure 3:**
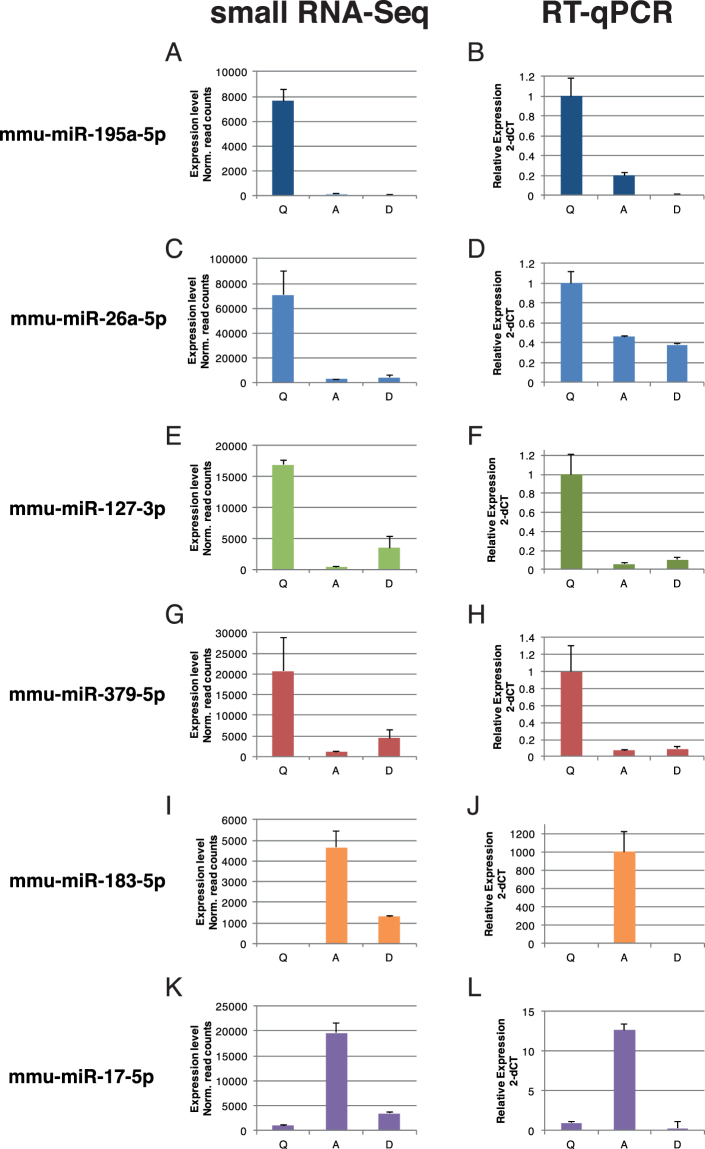
Validation of miRNA regulation on *in vivo* activated satellite cells Histogram presenting parallel expression measured by small-RNAseq following *in vitro* culture compared to *in vivo* activated satellite cells and isolated single muscle fibres. (**A**–**F**) The trend in expression was confirmed for 6 out 6 tested miRNAs, and only miR-26a did not show the same amplitude of deregulation on *in vivo* activated samples. (**G**–**J**) identical results were obtained for activation specific miRNAs, thus validating the miRNA-sequencing data using an *in vitro* activation paradigm. Normalization based on cell number allowed to confirm the higher expression level of many miRNAs during quiescence. Data are presented as Mean ± SEM.

To validate the expression of differentially regulated miRNAs during commitment *in vitro*, we isolated myogenic cells from (i) resting *Tibialis anterior* (TA) muscle, (ii) 3 days post-notexin (snake venom promotes myofibre lysis)^23^ injury of TA muscle, and (iii) dissociated *Extensor digitorium longus* (EDL) muscle fibres with stripped satellite cells. To compare the miRNA expression profiles by RT-qPCR across distinct cell states during myogenic commitment, we chose to normalize to the number of cells. Of the 6 differentially expressed miRNAs identified by sequencing that we validated, 5 showed both the expected trend and magnitude of dynamic expression (Fig. 3). For the remaining miRNA (miR-26b), the trend was similar but a less pronounced magnitude was observed. Additionally, we compared our sequencing dataset to the published profiling of miRNAs during *in vivo* activation obtained by RT-qPCR^12^. When focusing on the 228 miRNAs that were detected by both methods, we observed an overall concordance of data (Supplementary Fig. S4A). However, a number of miRNAs absent from the RT-qPCR dataset were detected, thereby complementing the miRNA profiles associated with the Quiescence/Activation transition states. Also, several miRNAs amplified by PCR were unambiguously absent from the sequencing dataset (Supplementary Fig. S4B). Taken together, these observations validated our *in vitro* model of satellite cell lineage progression and the quiescence/activation transition.

### A subset of miRNAs is disproportionally upregulated in quiescent satellite cells

Quiescent satellite cells have a reduced cytoplasmic to nuclear ratio, reduced metabolism, and lower levels of total mRNA and protein compared to activated and differentiated cells^24,25^. Previous reports stated that miRNAs were globally downregulated in human muscle stem cells^26^. We thus compared the miRNA and total RNA content in quiescent and activated satellite cells and found that the miRNA/total RNA ratio was in the same order of magnitude (T-test, *p-*value = 0.53; Supplementary Table S3). Moreover, given the per-cell normalization we used in our RT-qPCR assay, our analysis leads us to propose that tens of miRNAs have higher levels of expression in quiescent *vs*. activated satellite cells. Taken together, these findings suggest that the miRNAs over-expressed during quiescence are potent regulators that exert their effect in satellite cells.

### Comparative analysis of expressed miRNAs and Quiescence *vs*. Activated transcriptomes

Having identified a set of miRNAs specifically expressed during quiescence, we set out to assess their influence globally on the transcriptome by retrieving miRNA targets from Targetscan 7 database (http://www.targetscan.org)^27^. We selected mRNA targeted of the 123 miRNAs expressed in quiescent, but not in activated satellite cells, and obtained a list of 18,812 transcripts. We then compared their expression levels in quiescent *vs. in vivo*-activated satellite cells following an injury in 3 published datasets from 3 different laboratories^28–30^. Despite the lab-to-lab heterogeneity that could be observed in these theoretically identical datasets^30^, we found a significant down-regulation in quiescence of the targeted transcripts (Supplementary Fig. S5). Gene ontology (GO) enrichment analysis of the targets was however not informative due to the very high number of targets identified. We thus reduced this list to the target mRNA identified by applying more stringent criteria on putative targets from Targetscan with at least 1 conserved target site, and a Cumulative weighted context++ score <−0.2 (yielding to 4,814 putative targets), and conserved only those identified by 2 additional prediction tools (TarBase^31^ and miRDB)^32^. Once again, this set of 1,172 transcripts showed an overall downregulation in quiescent satellite cells (Supplementary Fig. S5). Given the high number of genes used, we filtered the results from the GO enrichment analysis (*FDR* <0.001 & enrichment >4). Surprisingly, most functions related to cardiac development regulation were identified in the dataset (Supplementary Table S4). Nevertheless, we also identified functions related to *skeletal muscle tissue development* (*p-*value < 5.9E-9), *regulation of ubiquitin-protein transferase activity* (*p-*value < 6.6E-4) and *somatic stem cell division* (*p-*value < 1.1E-4).

### A cluster of microRNAs on chromosome 12 is over-expressed in quiescent satellite cells

**Figure 4:**
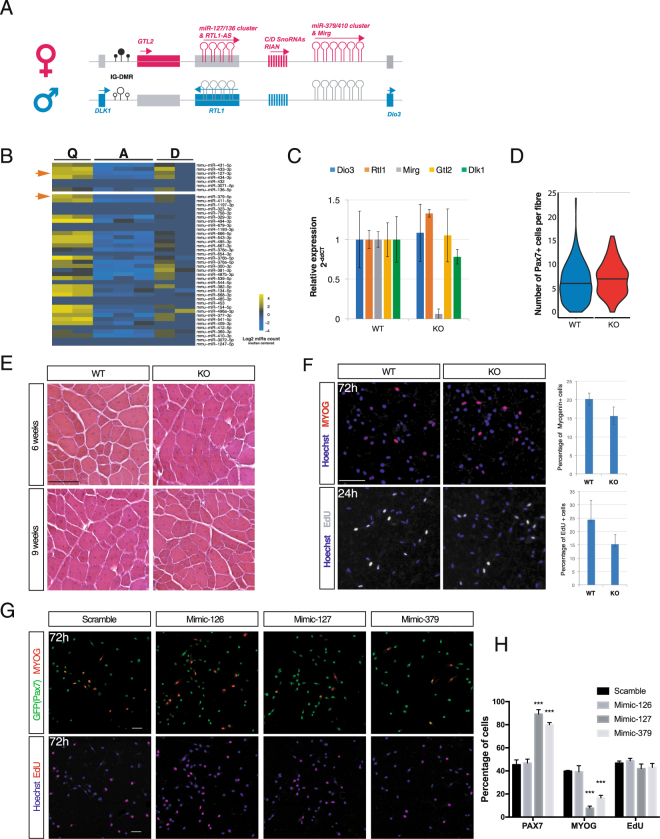
Functional analysis of miRNA from the *Dlk1-Dio3* locus expressed in quiescent satellite cells. (**A**) Schematic representation of the maternally expressed miR-127/miR-136 and miR-379/miR-410 gene cluster in the imprinted *Dlk1-Dio3* locus located on mouse chromosome 12. *Dlk1* and *Dio3* are expressed from paternally inherited chromosome. Drawing is not to scale. (**B**) Heatmap representing the expression level of miRNAs from the *Dlk1-Dio3* locus during lineage progression from quiescent to activated and differentiated satellite cells. The two miRNAs studied in more detail are highlighted with orange arrows. (**C**) Expression of coding and non-coding genes from the *Dlk1-Dio3* locus in control and mutant mice. The *Mirg* transcript, in the deleted region, is not detected in mutant satellite cells. (**D**) Counting of satellite cell numbers on EDL muscle fibres from mutant and WT mice; n = 6–7 mice/genotype; ≥15 fibres/mice. The bar in the violin plot represents the median. (**E**) H&E staining of TA muscle sections from WT and miR-379/410 mutants 6 and 9 weeks after Notexin injury of muscle. Scale bar, 100 μm. (**F**) Anti-Myog staining (top) and EdU reaction (bottom) in satellite cells isolated from *Dll1-Dio3* Control (WT) and mutant (KO) mice at 72 h and 24 h, respectively. Quantification of Myog+ and EdU+ cells at each time point revealed no difference between WT and KO satellite cells. Scale bar, 50 μm. (**G**) Anti-GFP, anti-Myog stainings (top) and EdU reaction (bottom) in satellite cells isolated from *Tg:Pax7-nGFP* mice 72 h after Mimic-127, Mimic-379 or Scramble control transfection. Scale bar: 25 μm. (**H**) Quantification of Pax7/GFP-positive, Myogenin-positive and EdU positive cells at 72 h following Mimic-127, Mimic-379 or Scramble transfection. Error bars indicate SD; n = 4 mice, ≥400 cells counted, 2 wells/condition.

A closer examination of the list of differentially expressed miRNAs identified tens of candidates that were located on chromosome 12 and belonged to two large clusters comprised in the *Dlk1-Dio3* locus (Fig. 4A). Notably, we found 5 and 25 miRNAs highly expressed in quiescence and lost upon activation of satellite cells from the *miR-127/miR-136* and *miR-379/miR-410* clusters, respectively (Fig. 4B). This locus is of major importance for the regulation of skeletal muscle hypertrophy^33^. Additionally, it was reported recently that the deletion of the *miR-379/544* locus induced a hypertrophy of fast-twitch muscles in neonates^34^. We thus chose to investigate the molecular function of these differentially expressed miRNAs specifically in satellite cells. To do so, we used a mouse model carrying a deletion of the maternally expressed *miR-379–410* cluster and *Mirg* non-coding gene^35^, to assess the consequences of these miRNAs on homeostasis and regeneration (Fig. 4C). We noted that there was no difference in numbers of Pax7^+^ satellite cells per fibre in resting EDL muscle of WT and KO mice (Fig. 4D). In addition, following Notexin injury of TA muscle, no overt regeneration phenotype was observed in KO mice (Fig. 4E). Finally, isolated satellite cells from both WT and KO resting muscles did not show any significant differences in their ability to proliferate, commit to myogenesis, differentiate, or self-renew as reserve cells in culture (Fig. 4F, data not shown). These findings raise the possibility that other compensatory miRNAs might act to regulate these transitions.

We then selected two candidate miRNAs with robust expression in quiescence for further analysis: miR-127-3p and miR-379-5p. These miRNAs that belong to the miR-127-3p and miR-379-5p clusters were also validated for their modulation of expression after *in vivo* activation (Fig. 3E–H). We assessed the potential impact of their expression on cell fate decisions by maintaining their expression level using transfected miR-mimics in satellite cells isolated from resting adult muscles. After 72 h culture of satellite cells, both miRNAs promoted a significant increase in the number of Pax7-expressing cells, whereas Myog-positive differentiating cells were reduced compared to control cells transfected with a scramble mimic (Fig. 4G–H). Following sustained expression of a third mimic miR-126-3p, no significant effect in these assays was noted in spite of the robust and specific expression of this miRNA in quiescent satellite cells and its previously demonstrated role in regulating quiescence in HSC^36^. These observations confirm the regulatory role of two miRNAs in regulating quiescence and self-renewal. However, the cell-cycle exit and proliferation of satellite cells was not altered by mimic-127 and −379, as measured by EdU incorporation (Fig. 4G–H). Given that Notch signalling is critical for satellite cell maintenance^37^, we examined the expression of miR-127-3p and miR-379-5p in *Rbpj* null satellite cells and found no significant alteration in their expression (data not shown). Interestingly, all expressed miRNAs from the *Dlk1-Dio3* locus except miR-379 were also reported to be highly expressed in long term HSCs^38^.

**Figure 5:**
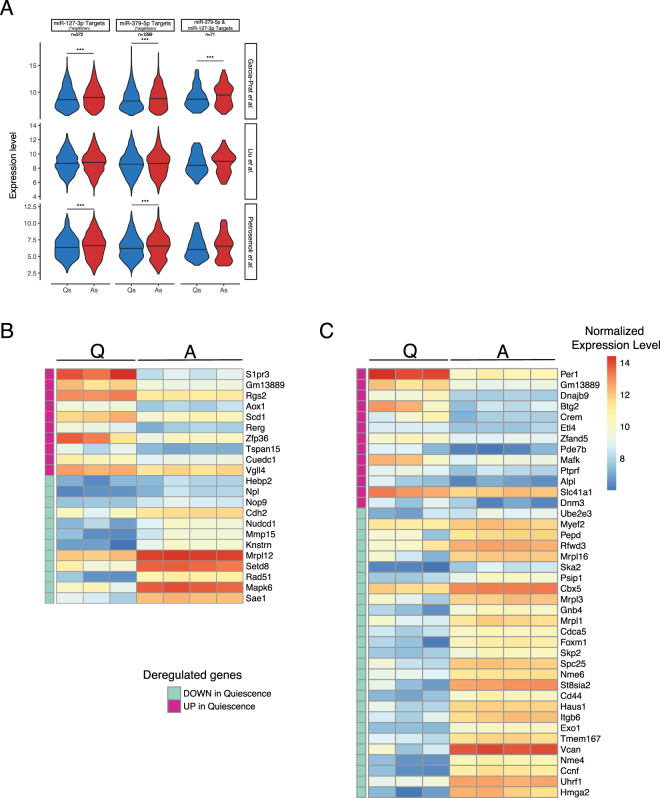
Analysis of the expression of putative targets of miR-127 and miR-379 in quiescent and activated satellite cells. (**A**) Expression level of putative mRNAs targets of miR-127 (left), miR-379 (middle) or both (right), predicted in Targetscan Db, in three datasets comparing quiescent vs. *in vivo* activated satellite cells. In two out of three datasets (Garcia-Prat *et al*. and Pietrosemoli *et al*.) transcripts targeted by either miRNA show significant downregulation in the transcriptome of quiescent (in blue) *vs*. activated satellite cells (in red) (Mann-Whitney test, *p-*value < 0.001). Targets common to the two datasets also showed a significant downregulation in quiescent cells in the first dataset. For all other comparisons, the same downregulation was observed on the medians, without reaching significance. This confirms an overall downregulation of the targets of these two miRNAs in quiescent satellite cells. Heatmaps of miR-127 (**B**) and miR-379 (**C**) putative targets also included in the 207 and 542 commonly up- and down-regulated genes identified in an integrated analysis of eight transcriptome datasets of quiescent satellite cells^30^. Expression levels of the corresponding genes in the dataset of Garcia-Prat *et al*. were used to define expression level. Down-regulated targets identified, that include epigenetic regulators (*SETD8*, HMGA2 and *CBX5*), or adhesion molecules (*CDH2*, *ITGB6* and *CD44)* are among candidates that could mediate the effect of miR-127 and miR-379.

We next set out to assess if the potential targets of these two miRNAs were modulated in quiescent *vs*. activated satellite cell transcriptomes. Targetscan Db predicted 572 and 1,299 targets for miR-127 and miR-379, respectively. Using the same transcriptome datasets as previously, we found an overall significant downregulation of the targets of quiescent miRNAs targets in quiescent satellite cells in two of them (Fig. 5A). The GO analysis on these target lists did however not yield a functional enrichment. To identify potential relevant targets for the regulation of quiescence, we thus crossed this list of potential targets to the 207 and 542 commonly up- and down-regulated genes identified in our recent analysis of eight transcriptome datasets of quiescent satellite cells^30^. Focusing on genes down-regulated in quiescence, we propose candidates whose knock-down might be mediating miR-127 and miR-379 control of quiescence and self-renewal, and notably epigenetic regulators (*SETD8*, *CBX5* and *HMGA2*) as well as adhesion/extracellular matrix genes (*CDH2*, *ITGB6* and *CD44*), or members of the ubiquitination pathway (*UBE2E3, UHRF1, RFWD3, CCNF, SKP2*).

## Discussion

In the framework of the present work, we provide the first open platform for analysis of small RNAs expressed during lineage progression of adult muscle stem cells. In this adult tissue stem cell paradigm, we did not observe the expression of piwi-RNAs that were reported to be expressed in germ cells^39^. However, some reads mapped to intronic regions that could constitute mirtrons and endo-siRNAs. Our data show that small RNAs expressed in the muscle lineage overwhelmingly correspond to microRNAs. Several reports have shed light on the regulation of miRNAs in muscle, but they detected only a limited number of small RNAs using RT-qPCR^12^ or miRNA microarrays^21^. The only miR-seq dataset reported did not include an isolated quiescent satellite cell sample, impeding the study of miRNA regulation in the transition states from quiescent to activated muscle stem cells^40^. Our comparisons with that report pointed to some discrepancies (*e.g*. absence of increase in miR-206 level during satellite cell activation, or absence of deregulation in miR-489 expression during early injury). However, our dataset was globally concordant with an RT-qPCR based analysis^12^.

We observed massive deregulation of miRNAs during the quiescence-activation transition in mouse satellite cells. This was unexpected given the low level of regulatory activity and reduced cytoplasmic content of quiescent muscle satellite cells. Instead, the relatively high number of miRNAs enriched during quiescence leads us to propose that cellular quiescence represents a poised state that is actively repressed by class-specific miRNAs. We showed experimentally that many miRNAs have a higher expression in quiescent cells compared to activated satellite cells underscoring the notion that the regulation of the quiescent state is an actively maintained process involving in part a large repertoire of miRNAs. Accordingly, the identification of miR-195/497 and miR-489 as regulators of the quiescence/activation transitions, and Notch signalling as a key mediator of the retention of satellite cells in their niche reinforces this notion^12,21,37,41^.

Our observations in the mouse are in clear contradiction with a report stating that miRNAs were all downregulated in human quiescent satellite cells which led to the proposal that quiescent cells represent minimal regulatory activity^26^. These discrepancies could be linked to a low number of miRNAs detected in the human study, that impeded the normalization and robustness of the data, or they might be related to *bona fide* species-specific differences. Interestingly, *Pax7*-positive quiescent satellite cells showed miR-27b expression, however miR-31 expression that was reported to be present in *Pax3*-expressing satellite cells was not expressed in *Pax7*-expressing satellite cells, thus pointing to potential differences in miRNA regulation between *Pax3*- and *Pax7*-expressing cells from trunk and limb, respectively^19,20^.

In this study, we identified novel miRNA candidates as potential regulators of cell state-specific transitions during myogenic lineage progression, and were interested to identify their influence on mRNA levels by combining several datasets of transcriptomes of quiescent satellite cells^28,29^. We observed an overall downregulation in quiescence of the several thousand mRNAs putatively targeted by quiescence miRNAs. These observations point to a collective control by miRNAs on the expression of specific mRNAs during these cell transitions. The number of miRNAs expressed in quiescence, and thus the number of potential targets identified complicated the global analysis of their function. Nevertheless, using a restricted list of potential targets generated by combining 3 prediction tools, we found Gene Ontologies associated with regulation of muscle development, including skeletal muscle, but more largely of cardiac development.

Having identified the overexpression of tens of miRNAs from two clusters located in the *Dlk1-Dio3* locus in quiescent satellite cells, we investigated the impact of the deletion of the miR-379/410 cluster *in vivo*. Our observation that this resulted in no overt regeneration phenotype or modulation of cell fates of isolated satellite cells leads us to speculate that other miRNAs have overlapping functions with this cluster. In contrast, functional analysis by sustained expression of two miRNAs from the miR-127 and miR-379 loci resulted in a robust phenotype corresponding to an increase in Pax7 expression and reduced commitment towards differentiation, thereby indicating that these miRNAs promote the stem cell state. These findings suggest that these miRNAs play key roles in regulating satellite cell quiescence in adult resting muscles.

In their *in vivo* genetic inactivation study of a part of the same miR-379/410 cluster, Gao *et al*. linked their phenotype to the targeting of *Dlk1* expression level by miR-329^34^. Our results appear to differ from this interpretation given that the phenotype induced by miR-127 and miR-379 overexpression do not correspond to results obtained after conditional deletion of *Dlk1* in satellite cells^42^. Specifically, we did not observe altered proliferation, but instead increased self-renewal and decreased differentiation of satellite cells. The absence of a regeneration phenotype in the miR-379/410 mutant mice could be explained by compensations by other miRNAs expressed in quiescence targeting common targets, or other compensatory mechanisms during development following the germline deletion. The implication of a re-expression of the paternal allele of the miRNA-cluster is however unlikely, as no expression of *Mirg*, a maternally expressed ncRNAs that overlaps with the miR-379/411 cluster, was detected in satellite cells in mutant mice. Unfortunately, the size of the locus to be deleted precludes the use of a cell type specific inducible Cre-recombinase under the control of *Pax7* to study the role of these miRNAs in more detail.

Future work will be required in gain or loss of function experiments to uncover the molecular function of additional differentially expressed miRNAs, and to identify their relevant targets in the context of induction and maintenance of quiescence, beyond the pivotal role of miR-489 and miR-195/497 already noted in *Pax7*-positive cells. In addition, identifying the signalling pathways upstream of these miRNAs will shed light on this tightly regulated biological process.

In summary, our findings that a relatively significant variety of miRNAs are dedicated to negotiate the quiescence to activation states of muscle stem cells suggests that quiescence is actively repressed by this class of regulators, but in a poised state. These results can impact on our views of genetic and epigenetic regulation of quiescence and how this critical cell state is regulated in homeostasis and trauma.

## Methods

### Mice and flow cytometry of satellite cells

Quiescent muscle stem cells were collected from adult *Tg:Pax7-nGFP* mice as described previously. Six-weeks old male mice were sacrificed by cervical dislocation, and their limb muscle were dissected, minced and digested in collagenase 0.1% and trypsin 0.25% at 37 °C under gentle agitation. Cells were collected in serum-containing medium and subjected to isolation by FACS based on positive GFP-fluorescence and negative Propidium Iodide fluorescence (10 µg/ml; Sigma-Aldrich). *In vivo* activated satellite cells were collected by FACS from regenerating injured muscle. The *Tibialis anterior* (TA) muscles of 6-week-old *Tg:Pax7-nGFP* mice were injured by intramuscular injection of the snake venom Notexin (10 μl, 10 mM; Latoxan) under anesthesia (0.5% Imalgene/2% Rompun) as described^43^. Four days after injury, regenerating TA muscles were dissected, dissociated and cells were isolated as aforementioned. Adult limb muscles from miR-379/410 mutant mice were dissected, minced and incubated with a mix of Dispase II (Roche) 3 U/ml, Collagenase A (Roche) 100 ug/ml and DNase I (Roche) 10 mg/ml in Hank’s Balanced Salt Solution (HBSS, Gibco) at 37 °C under gentle agitation for 2 h. Satellite cells were sorted by a combination of cell surface markers: α7-integrin-APC (Ablab, clone R2F2, 1/1000), CD34-e450 (eBioscience, clone RAM34, 1/50), Sca1-Pe-Cy7 (eBioscience, clone D7, 1/400), CD45-Pe-Cy7 (eBioscience, clone 30-F11, 1/100) and CD31-PE (BD, clone MEC13.3, 1/50) as described^44,45^. All experiments with animals were performed under conditions established by the European Community and approved by the Ethics Committee at Institut Pasteur, and the French Ministry.

### Myofibre isolation

Single myofibres from *Extensor digitorum longus* (EDL) muscle from 6-week-old adult *Tg:Pax7-nGFP* mice were dissociated as described^46^. Briefly, EDL muscles were dissected and incubated in 0.1% w/v collagenase (Sigma, C0130)/DMEM for 1 h in a 37 °C shaking water bath. Following mechanical release, individual myofibres were either processed for RNA extraction to validate the RNA sequencing in differentiation conditions, or directly fixed in 4% paraformaldehyde (PFA, Electron Microscopy Sciences).

### Satellite cell culture and differentiation

Freshly isolated satellite cells were seeded at 3,000 cells/cm^2^ in growth medium containing 1:1 DMEM:MCDB (Gibco and Sigma-Aldrich, respectively) supplemented with 20% serum FBS (Gibco) and 1% Ultroser G (Pall) on Matrigel coated flasks (BD Biosciences) and cultured in an incubator under physiological oxygen pressure (37 °C, 6.5% CO_2_, 3% O_2_). To assess proliferation, cells were pulsed with the thymidine analogue 5-ethynyl-2′-deoxyuridine (EdU, 1 × 10^−6^ M), 2 h prior to fixation (ThermoFisher Click-iT Plus EdU kit, C10640). For differentiation experiments, 60 h after plating, medium was replaced to remove Ultroser G, and cells were cultured for a total of 7 days to reach early differentiation.

### Satellite cell transfection

Freshly isolated satellite cells from *Tg:Pax7-nGFP* were transfected in suspension immediately after FACS with miRIDIAN microRNA hsa-miR-126-3p mimic (UCGUACCGUGAGUAAUAAUGCG, Dharmacon, C310987), mmu-miR-379-5p (UGGUAGACUAUGGAACGUAGG, Dharmacon, C310603), mmu-miR-127-3p (UCGGAUCCGUCUGAGCUUGGCU, Dharmacon, C310397) and Scramble Control#1 (UCACAACCUCCUAGAAAGAGUAGA; Dharmacon, CN-001000) at 200 nM final concentration using Lipofectamine 2000 (ThermoFisher) in Opti-MEM (Gibco). Four hours after transfection, 3 volumes of fresh growth medium were added and cells were cultured for the indicated time.

### Antibodies and immunostainings

Cells and myofibers were fixed in 4% PFA for 5 min, permeabilised 5 min in 0.5% Triton-X100 (Sigma-Aldrich) and blocked in 10% normal goat-serum (Gibco) for 30 min at RT. Cells and fibres were then incubated with primary antibodies (Chick GFP, Abcam13970,1/2000; mouse Pax7, DHSB, 1/40; rabbit Myod, Santa Cruz, 1/200; mouse Myogenin (F5D), DHSB, 1/40) overnight at 4 °C. Samples were washed with 1X PBS three times and incubated with Alexa-conjugated secondary antibodies (Life Technologies, 1/1000) and Hoechst (Life Technologies, 1/10000) for 45 min at RT. EdU staining was chemically revealed using the Click-iT Plus kit according to manufacturer’s recommendations (Life Technologies, C10640). Images were acquired using an upright fluorescent microscope (Zeiss).

### Histology

Isolated TA muscles were fixed in PFA 2%/0.2% Triton/PBS 1X for 2 h at 4 °C, washed in PBS 1X overnight at 4 °C and incubated in 20% sucrose/PBS 1X for an additional 12 h. TA muscles were embedded in OCT and frozen in liquid nitrogen prior to cryosectioning (10 µm) and stained with Hematoxylin/Eosin. Images were acquired with Zeiss Axioscan microscope.

### Small RNA extraction, deep sequencing and RT-qPCR

For small RNA collection, quiescent cells were directly sorted into Trizol-LS reagent (Invitrogen), and *in-vitro* cultured cells (activated at 60 hours and differentiated at 7 days) collected in Qiazol reagent (QIagen). Total RNA was subsequently purified using the miRNeasy Mini Kit following the manufacturer's instructions (Qiagen). Ten micrograms of total RNA obtained from several animals for the quiescent samples, were used for each biological replicate prepared for deep sequencing (*i.e*. 2 replicates for the quiescent and differentiated samples, and 3 replicates for the *in vitro* activated sample). For RT-qPCR validations all samples were extracted using the same methods (Trizol LS after FACS for quiescent and *in-vivo* activated satellite cells; Qiazol for isolated single fibres).

For validations, reverse transcription was performed on RNA amount corresponding to fixed absolute number of cells for quiescent and activated SC (i.e. 25,000 cells per RT) in order to be compared. For differentiated muscle fibres, the amount of RNA used in the reverse transcription and following PCR was measured to be equivalent to the activated cells. Reverse transcription of miRNAs was performed on total RNA using the miRCURY LNA Universal RT-PCR system following the manufacturer’s instructions (Exiqon). Quantitative PCR was performed using SYBR Green based mix (Exiqon) and LNA™ PCR primer set (Exiqon) targeting mmu-miR-127-3p (Ref.204048), mmu-miR-379 (Ref.204296), mmu-mir-26a (Ref.204724), mmu-mir-195 (Ref.204186), mmu-miR-183 (Ref.204652), mmu-mir-17 (Ref.204108), U6 snRNA (Ref.203907) and RNU5G (Ref.203908). Analysis was performed using the 2^−∆CT^ method^47^.

### Total mRNA extraction and RT-qPCR

Total mRNAs were isolated using (Qiagen RNAeasy® Micro Kit) and reverse transcribed using SuperScriptIII^®^ enzyme (Invitrogen, 18080093). The remaining RNAs were degraded by incubation for 20 min at 37 °C with RNase H endonuclease (Roche). Expression of mature mRNAs was assessed with SYBR green master mix (Roche) and analyses were performed using the 2^−∆∆CT^ method^47^. All RT-qPCR analyses were normalized with *Tbp* and *Rpl13*. Specific forward and reverse primers used for RT-qPCR are listed in Supplementary Table S5.

### Total RNA extraction and Bioanalyser analysis

Total RNAs, including small RNAs, were isolated from quiescent and 48 h *in vitro* activated satellite cells using Direct-zol RNA Microprep (Zymo Research) according to manufacturer’s recommendations. Total RNA and small RNA ratio were quantified using the Agilent 2100 Bioanalyzer PicoChip and SmallChip and analyzed with 2100 Expert software.

### Size fractionation of RNAs

For each biological replicate, 10 µg of total RNA (in 10 µl) were mixed with 10 µl of 2X TBE-Urea Sample Buffer (Invitrogen) and loaded in a well of a 15% polyacrylamide TBE-urea gel (Biorad). After migration, the gel was soaked in a SYBR gold (Invitrogen) solution, and imaged on a Dark Reader transilluminator. The 18–35 nucleotide region was cut using a scalpel for each sample, and the RNA eluted in 300 µl of 0.3 M NaCl solution under rotation for 4 hours at room temperature. The eluate was transferred together with gel debris onto a Spin X cellulose acetate filter (VWR) and centrifuged for 2 minutes at 12,000 xg. Small RNAs were finally precipitated by addition of 1 μl of glycogen (Invitrogen) and 750 μl of room temperature 100% ethanol followed by an incubation at −80 °C for 30 min, and centrifugation for 25 min at 14,000 rpm and + 4 °C. The pellet was washed with 750 µl 75% Ethanol, dried and resuspended in 5 µl ultrapure water with 0.5 µl of RNAseOUT (Invitrogen).

### Library preparation for small RNA-seq

Small RNAs purified on gel were mixed to 1 µl of 10 µM pre-adenylated 3′ Illumina linker V1.5 (5′-rAppATCTCGTATGCCGTCTTCTGCTTG/3ddC/-3′), denatured for 2 min at 70 °C, and further mixed with 1 µl of 10x T4 RNA-Ligase Truncated Reaction buffer, 0.8 µl 100 mM MgCl_2_, 0.5 µl RNaseOut and 1.5 µl of T4 RNA Ligase 2 truncated (New England Biolabs). Ligation was performed at 22 °C for 1 h. Then, 0.5 µl of 5′-RNA adapter (5′-r(GUU CAG AGU UCU ACA GUC CGA CGA UC)-3′), 1 µl of 10 mM ATP and 1 µl T4 RNA ligase (Ambion) were added, and ligation was performed at 20 °C for 6 h. Adaptor ligated RNA in a volume of 4 µl were then mixed with 1 µl of 20 μM Solexa RT primer (5′-CAA GCA GAA GAC GGC ATA CGA-3′) and denatured at 70 °C and cooled on ice. Reverse transcription was then performed after addition of 2 µl 5x first strand buffer (Invitrogen), 0.5 µl of 12.5 mM dNTP mix, 1 µl of 100 mM DTT, 0.5 µl_ RNase OUT and 1 µl SuperScript III Reverse Transcriptase (Invitrogen) at 50 °C for 1 h, followed by 10 min at 70 °C. The obtained cDNA was PCR-amplified by addition of 27 µl Ultra-pure water, 10 µl 5× Phusion-HF buffer, 1 µl of 25 µM Forward Primer (5′-AAT GAT ACG GCG ACC ACC GAC AGG TTC AGA GTT CTA CAG TCC GA-3′), 1 µl of 25 µM reverse Primer (5′-CAA GCA GAA GAC GGC ATA CGA-3′), 0.5 µl of 25 mM dNTP mix, and 0.5 µl Phusion DNA Polymerase (Finnzymes) using 12 cycles 98 °C 10 sec/60 °C 30 sec/72 °C 15 sec. The library was finally purified on a 5% TBE PAGE gel, by cutting the region corresponding to the 92–106 bp (the ligated linkers corresponding to a 73 bp band visible on the gel). The gel was crushed by centrifugation and eluted in 1X Elution buffer (Illumina) by rotation for 2 h at RT. The eluate was cleared using a Spin-X column and precipitated after addition of 1 µl of glycogen, 10 µl of 3 M NaOAc and 325 µl of −20 °C 100% ethanol, followed by centrifugation for 20 min at 14,000 rpm. After washing, the pellet was resuspended in 1 ml dH_2_O. Finally, the sample was diluted to 10 nM and submitted to sequencing on a Solexa GA-IIX at the core sequencing facility.

### Bioinformatic analysis and statistics

Analysis of the microRNAs expression was performed from fastq raw files using the Galaxy Mississipi tool suite (https://mississippi.snv.jussieu.fr) provided by ARTbio bioinformatics analysis facility (Sorbonne Universités, UPMC Univ. Paris 06, CNRS FR3631 Institut de Biologie Paris Seine, Paris, France). Briefly, after trimming of adapters, reads were mapped on Mus musculus mature miRNA sequences from miRbase 19 using sRbowtie. Normalization of miRNAs counts and differential analysis was further performed using DESeq. 2 using replicate samples. MicroRNAs with a corrected *p-v*alue < 0.001 (Benjamini-Hochberg method) were considered as differentially expressed. Annotation of reads were performed by sequential alignment of reads on collections of annotated RNA sequences including ribosomal, mitochondrial RNA, exonic and intronic mRNA, piRNA and miRNAs as previously described^48^.

K-means clustering of differentially expressed miRNAs was performed using Cluster 3.0 (available at bonsai.hgc.jp/~mdehoon/software/cluster/software.htm) using normalized read count as input. After a step of median centering of expression level for each miRNA, clustering was perform using centered Pearson correlation with 1,000 iterations. The corresponding heatmap was generated using JavaTreeView (http://jtreeview.sourceforge.net).

For the mRNA/miRNA correlation analyses, data from Targetscan 7 database were filtered using in-house scripts using stringency in the number of sites and Total context++ score^27^. For correlation with mRNA expression level, the publicly available datasets GSE47177 and GSE70376 were obtained from the Gene Expression Omnibus (www.ncbi.nlm.nih.gov/geo). Comparisons of expression level between the groups of transcripts in the different satellite cell states (*i.e*. quiescent or activated *in vivo* 3 days post injury) were performed using a Mann-Whitney test. All statistical tests were performed in R.

### Data Availability

The small RNA-seq data generated and analysed during the current study have been deposited in the ArrayExpress database at EMBL-EBI under accession number E-MTAB-5955 [https://www.ebi.ac.uk/arrayexpress/experiments/E-MTAB-5955].

## Electronic supplementary material

Supplementary Figures

Supplementary Table S1

Supplementary Table S2

Supplementary Table S4
