# Supplementary material for: Small-RNA sequencing identifies dynamic microRNA deregulation during skeletal muscle lineage progression
Source: Sci Rep. 2018 Mar 9;8:4208. doi: 10.1038/s41598-018-21991-w (PMC5844870; doi:10.1038/s41598-018-21991-w)
Supplement: Supplementary file 1 — Supplementary Figures [file 41598_2018_21991_MOESM1_ESM.pdf]

Supplementary Information – Supplementary Figures

Small-RNA sequencing identifies dynamic microRNA deregulation during skeletal muscle lineage progression

David Castel[1#], Meryem B. Baghdadi[1,2,3], Sébastien Mella[1,2], Barbara Gayraud-Morel[1,2], Virginie Marty[4], Jérôme Cavaillé[4], Christophe Antoniewski[5,6] & Shahragim Tajbakhsh[1,2*]

[1] Stem Cells and Development, Department of Developmental & Stem Cell Biology, Institut Pasteur, Paris 75015, France

[2] CNRS UMR 3738, Institut Pasteur, Paris 75015, France.

[3] Sorbonne Universités, UPMC, University of Paris 06, IFD-ED 515, 4 Place Jussieu, Paris 75252, France.

[4] Laboratoire de Biologie Moléculaire Eucaryote, Centre de Biologie Intégrative (CBI), Université de Toulouse, CNRS, UPS, 31000 Toulouse, France

[5] Sorbonne Universités, Université Pierre et Marie Curie (UPMC), CNRS, Institut de Biologie Paris Seine (IBPS), Developmental Biology Department, Paris, France.

[6] Sorbonne Universités, Université Pierre et Marie Curie (UPMC), CNRS, Institut de Biologie Paris Seine (IBPS), ARTbio Bioinformatics Analysis Facility, Paris, France

[#]present address: Département de Cancérologie de l'Enfant et de l'Adolescent & UMR8203 "Vectorologie et Thérapeutiques Anticancéreuses", CNRS, Gustave Roussy, Univ. Paris-Sud, Université Paris-Saclay, 94805, Villejuif, France

*Correspondence: shahragim.tajbakhsh@pasteur.fr

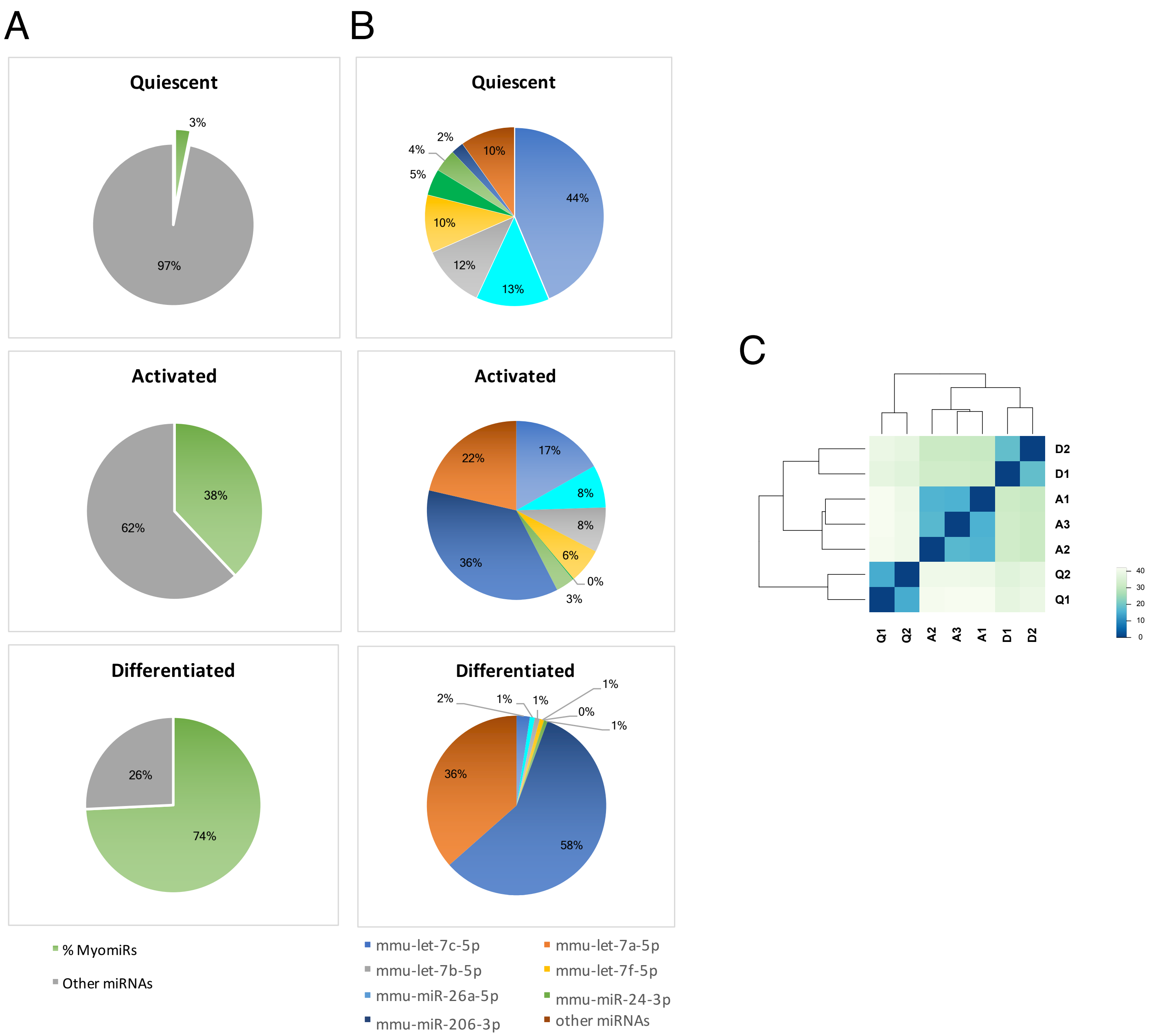

Figure S1. Overall comparison of miRNAs during lineage myogenic progression.

(A) Proportion of myomiRs among the miRNAs detected in the distinct cellular states analysed. MyomiRs represent as low as 3% in quiescent cells, but up to 74% of sequences in differentiated cells.
(B) Comparison of expression of the seven most abundant miRNAs in quiescence during myogenic lineage progression.
Pie-charts display the percentage of reads of the mostly expressed miRNAs in differentiated cells in all 3 biological conditions. A wide variety of miRNAs are expressed in quiescent cells, whereas some miRNAs such as mir-21 (middle) or miR-1 and miR-206 account for an increasing part of the detected miRNAs (around 60% of reads in differentiated samples). This points to wide modulation of miRNA expression patterns, and underscored necessity of robust normalization of the data.
(C) Assessment of overall similarities and dissimilarities between biological samples.
An unsupervised hierarchical clustering of biological samples was performed using the euclidian distance metrics based on rlog-transformed miRNAs expression counts. The heatmap displays the similarities between samples with dark blue color, together with a dendrogram. All samples regrouped according to each of the 3 biological conditions (quiescent, activated or differentiated) confirming the similitude of biological replicates. Activated and differentiated samples appeared more closely related than quiescent cells.

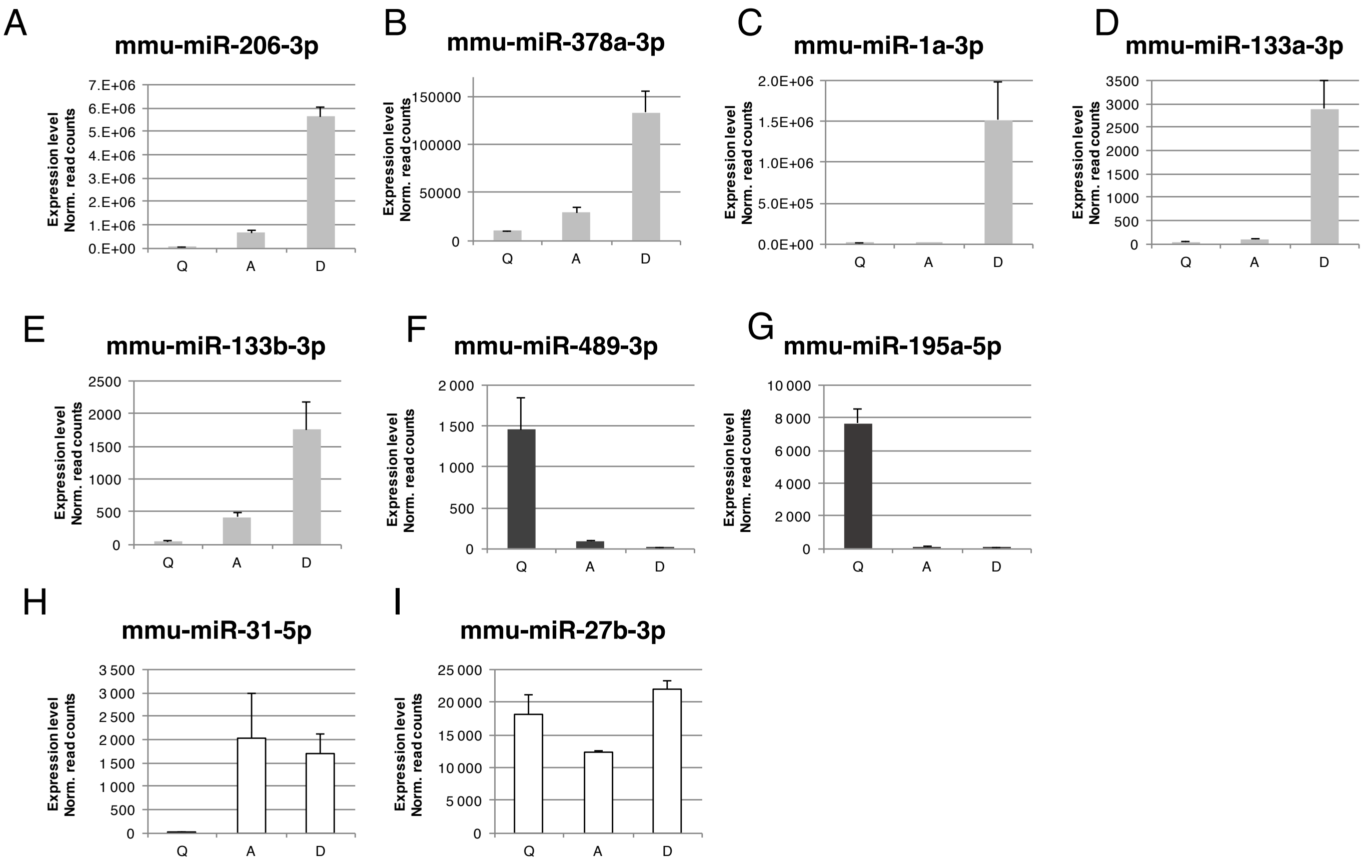

Figure S2. Expression profile of miRNAs previously identified in the muscle lineage.
Histogram of normalized miRNAs counts measured in quiescent, activated and differentiated satellite cells.
(A-E) Canonical myomiRs, *i.e.* miR-206, miR-378, miR-1 and miR-133, previously identified as upregulated during activation and differentiation showed a robust induction in the small RNA-seq dataset.
(F,G) miR-489 and miR-195, previously associated with satellite cell quiescence were specifically expressed in quiescent samples.
(H, I) miR-31 and miR-27b expression profiles were discordant with *Pax7*-expressing satellite cells showing a down-regulation, or a high expression in quiescent cells, respectively.

Castel *et al.* Supplementary Figure S3

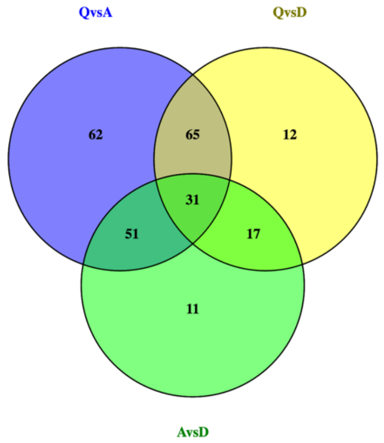

Figure S3. Comparison of differentially expressed miRNAs between different cellular states.
Many miRNAs identified as regulated during lineage progression are associated with the quiescent condition. Conversely, most miRNAs that were deregulated between activated and differentiated myoblasts were also deregulated between quiescent and activated satellite cells, or quiescent satellite cells and differentiated cells.

Castel *et al.* Supplementary Figure S4

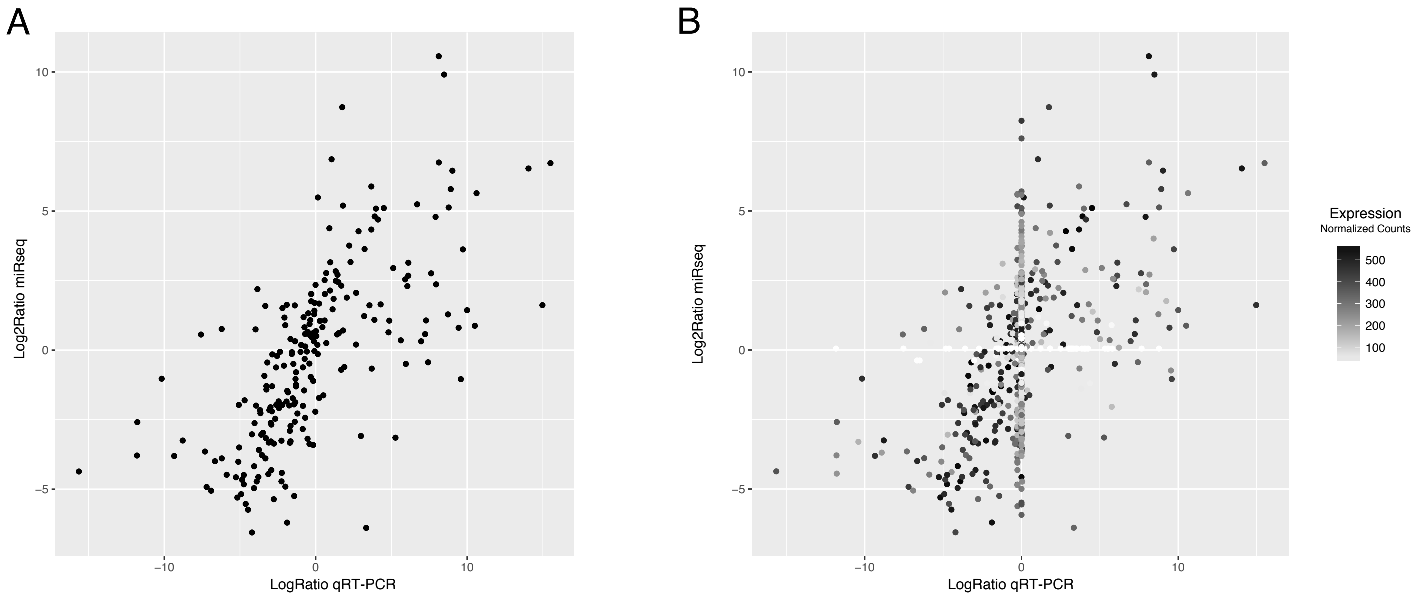

Figure S4. Comparison of data from miR-seq and from previously reported RT-qPCR profiling.
A) The log-transformed ratios of [Activated/Quiescent] expression levels were plotted to examine the concordance of data between the present dataset and those reported previously[12]. Data were filtered for the 228 miRNAs detected by both methods, to highlight the identical trend in expression observed in the two datasets.
B) The same data as in panel A but unfiltered. Circles were colored from white to black according to the average expression level in the miRseq dataset. A subset of miRNAs distributing on the X-axis (white circles) were not detected in the sequencing dataset compared to the PCR experiment, constituting potential false-positives. Conversely, a significant subset of miRNAs that were not detected in the RT-qPCR experiment, were detected in the sequencing dataset and distributed along the Y-axis.

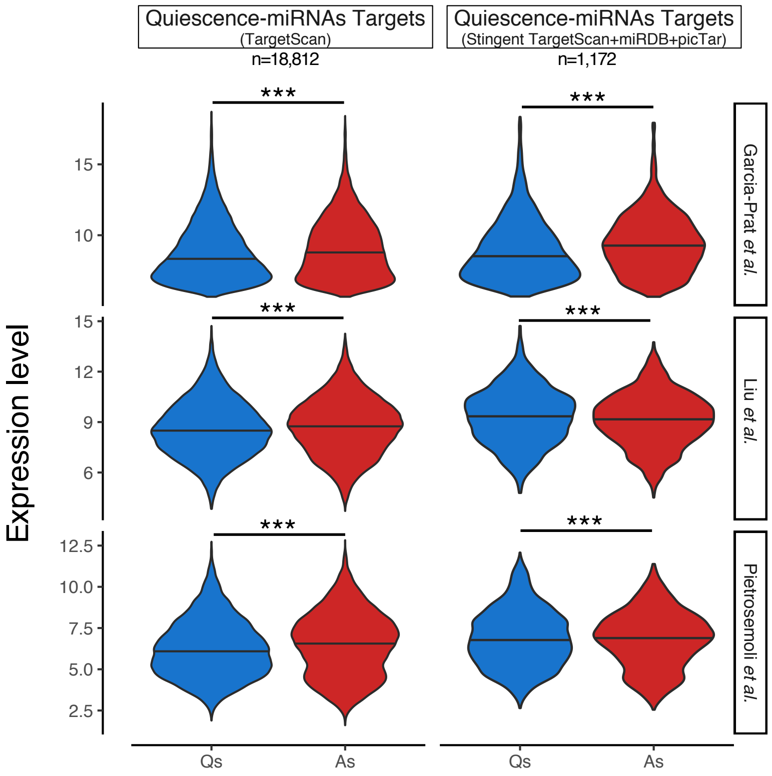

Figure S5. Comparative analysis of differentially expressed miRNAs and quiescent *vs.* activated satellite cells transcriptomes.

All miRNA targets predicted from Targetscan (left), or more stringently selected targets from Targetscan (≥1 conserved target site(s) & a Cumulative weighted context++ score<-0.2) also predicted in miRDB and picTar DB (right) were trimmed from the corresponding prediction databases. All transcripts targeted by the 123 miRNAs expressed in quiescent but not activated satellite cells were selected (n= 18,812 and 1,172 targets, respectively). Violin plots display the expression level in 3 distinct transcriptome datasets of the identified targets in quiescent (blue) *vs. in vivo* activated satellite cells 3 days post-injury of TA muscle (red). For the exhaustive set of targets of quiescent miRNAs (left), a significant down-regulation of transcripts was observed in all 3 datasets. For the reduced set of targets, a down-regulation occurred in 2 out of 3 datasets, with a contrary trend observed for the Liu *et al.* data. (Mann & Wittney test; *p*-value<0.001).

Supplementary Table S1. Normalized miRNAs expression data in quiescent, activated and differentiated satellite cells.

Supplementary Table S2. K-means clusters of differentially-expressed miRNAs identified in quiescent, activated and differentiated satellite cells.

Supplementary Table S4. Gene Ontology enrichment analysis on putative mRNA targets of miRNAs expressed in quiescent satellite cells

	QUIESCENT Satellite Cells	ACTIVATED Satellite Cells
**total RNA concentration (pg/ul)**	1174	3829
	782	3480
	NA	5792
**miRNA concentration (pg/µl)**	*37*	*314*
	52	*205*
	NA	*307*
**Ratio miR/totalRNA**	0.03	0.08
	0.07	0.06
	NA	0.05
**Mean ratio ±SD**	**0.049 ±0.025**	**0.065 +0.015**

Supplementary Table S3. Comparison of total RNA and miRNA content in Quiescent *vs*. Activated satellite cells.

Total RNA from *in vivo* isolated Quiescent and *in vitro* activated satellite cells were extracted from distinct mice and analysed using the Bioanalyzer Total RNA pico assay and Bioanalyzer Small RNA assay to generate total RNA and miRNA concentrations, respectively. For each analysed sample, the miRNA/totRNA ratio was calculated. Average value indicates that the miR/totRNA ratio is in the same order of magnitude (T-test, *p*-value=0.53). Despite a lower per cell content of total RNA in quiescent vs. activated satellite cells, the pool of expressed miRNAs varies proportionally.

Supplementary Table S5

PRIMER NAME	SEQUENCE
Dlk1_FW	CGGGAAATTCTGCGAAATAG
Dlk1_REV	TGTGCAGGAGCATTCGTACT
Rtl1_FW	GGCTGTTCTGCTCTACGAGG
Rtl1_REV	AAATTCGTCATCCGCCACCT
Mirg_FW	GACGGTGCAGACACCTTGTA
Mirg_REV	ATCCTGCCCACCAATGAAGG
Dio3_FW	GGAGTCTCCCGCCAATTCAA
Dio3_REV	CTTCTCCGACCACCAACCTC
Gtl2_FW	TGGGGATGGGTCTCTAGGTG
Gtl2_REV	CCACTGACCCACAGTAACCC
Tbp_FW	ATCCCAAGCGATTTGCTG
Tbp_REV	CCTGTGCACACCATTTTTCC
Rpl13a_FW	GTGGTCCCTGCTGCTCTCAAG
Rpl13a_REV	CGATAGTGCATCTTGGCCTTTT

Supplementary Table S5. List of primers used in the study
